# Imaging evaluation of the benign and malignant lesions of the floor of the mouth: Pictorial review

**DOI:** 10.4102/sajr.v27i1.2677

**Published:** 2023-08-31

**Authors:** Ashim K. Lahiri, Charles R. Daultrey

**Affiliations:** 1 Department of Radiology, Worcestershire Acute Hospitals NHS Trust, Worcester, United Kingdom; 2 Department of ENT and Head and Neck Surgery, Worcestershire Acute Hospitals NHS Trust, Worcester, United Kingdom

**Keywords:** floor of mouth, sublingual space, mylohyoid, computed tomography, CT, MRI

## Abstract

The floor of the mouth is an important anatomical region of the oral cavity where primary benign and malignant disease processes can originate or secondary pathologies can extend into adjacent spaces. Knowledge of the anatomy is crucial for accurate localisation of pathology and understanding the spread of disease. The sublingual space is the dominant component of the floor of the mouth, bounded inferiorly by the mylohyoid muscle that separates it from the submandibular space. Imaging is immensely important to characterise and map the extent of disease, considering the fact that the bulk of the disease may be submucosal and not visible on clinical inspection.

**Contribution:**

The floor of the mouth is a complex anatomical region for radiological evaluation. The purpose of this pictorial review is to present an understanding of the relevant anatomy and to demonstrate the role and appropriate application of different imaging modalities. This article highlights the imaging spectrum of a wide range of various benign conditions including normal variants and a variety of malignant lesions at different tumour stages, with an aim to establish the correct diagnosis, avoid misinterpretation and help in treatment planning.

## Introduction

Radiological evaluation of the floor of the mouth (FOM), an anatomical compartment of the oral cavity, is complex and challenging.^1^ The region harbours different types of tissues, including salivary glands, ducts, mucosa, submucosal soft tissues and the bony mandible and can be associated with a wide range of diseases, including congenital, inflammatory, infective, benign and malignant tumours and pseudolesions.^2,3^

Imaging is of immense value for evaluating submucosal disease of the FOM, which may not be completely obvious on clinical inspection.^2,4^ Ultrasound, CT, MRI and ^18^F-fluorodeoxyglucose positron emission tomography/computed tomography (^18^F-FDG PET/CT) imaging are crucial and complementary imaging techniques in diagnosis, disease mapping, treatment planning and surveillance.^2,3,4,5^

This manuscript reviews the pertinent anatomy, clinical contexts and distinct imaging characteristics of different pathologies and pseudolesions affecting the FOM.

## Anatomy

**FIGURE 1:**
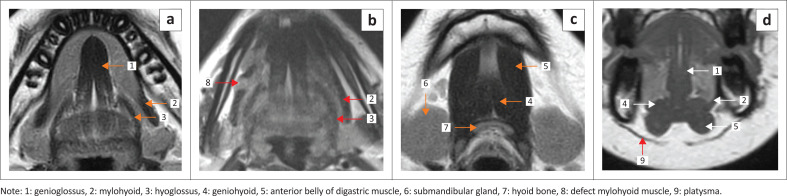
MRI anatomy of the floor of the mouth on T1 weighted (T1W) images indicating the muscles of the floor of the mouth and the extrinsic muscles of the tongue. Inferior axial (a), mid axial (b), superior axial (c) and coronal (d).

**FIGURE 2:**
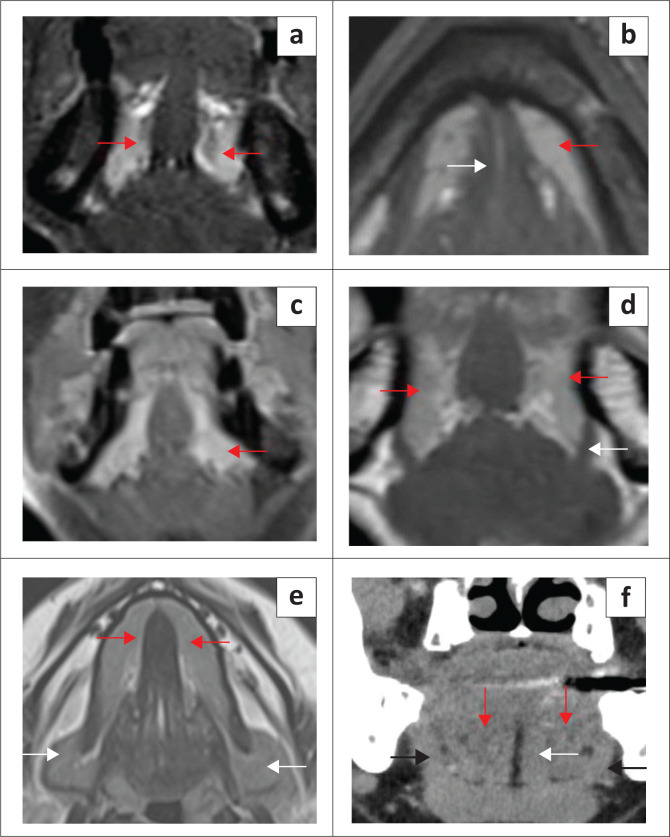
Anatomy of the sublingual space. T2 fat-suppressed coronal (a), T2 axial (b), T1 contrast-enhanced fat-suppressed (c) and T1 coronal without fat-suppression (d) show the sublingual glands (red arrows), mylohyoid muscle (d, white arrow), geniohyoid (b, white arrow). T2 axial (e) demonstrates the deep part of submandibular glands (white arrows) in floor of the mouth (FOM), posterior to the mylohyoid muscles and sublingual glands (red arrows). CT coronal (f) shows the mixed fat density of sublingual glands (red arrows) between the genioglossus (white arrow) and mylohyoid muscles (black arrows).

The FOM is an important region of the oral cavity, located inferior to the oral tongue, bound anteriorly and laterally by the inner mandibular gingiva and inferiorly by the mylohyoid and geniohyoid muscles (Figure 1 and Figure 2). The posterior margin of the FOM corresponds to the attachment of the anterior tonsillar pillar to the tongue.

The sublingual space (SLS) is the major dominant deep neck anatomical space and is also referred to as the FOM, divided into bilateral spaces by the frenulum of the tongue.^1,2,3,4^ The SLS lies lateral to the genioglossus and geniohyoid muscles and superior to the mylohyoid muscle (Figure 1d and Figure 2). The contents of the SLS are fat, the sublingual glands, the lingual nerve (branch of V3 trigeminal nerve), lingual vessels, branches of the glossopharyngeal and hypoglossal nerves (IX and XII cranial nerves), the superior most part of the submandibular gland, Wharton’s duct (submandibular) and part of the hyoglossus muscle.^3,5,6^ The SLSs communicate freely with each other anteriorly and communicate with the submandibular spaces inferiorly through the gap between the free posterior margin of the mylohyoid and hyoglossus muscles^4,6,7^ (Figure 1b and Figure 2e). Extension of disease can occur from the submandibular to the parpharyngeal space through the communicating channels.^7,8^

The sublingual glands are formed by smaller glands and are drained by short ducts in the FOM/SLSs. These glands are best visualised on T2-weighted short-tau inversion recovery (T2-STIR) and post-contrast MRI^8^ (Figure 2a, c). The submandibular (Wharton) ducts pierce the mylohyoid muscles and open in the FOM, on either side of the frenulum. The duct opening can be occluded by calculi or tumours and patients can present with features of the obstructive submandibular adenitis.

The mylohyoid muscle is an important anatomical landmark in the FOM; it separates the SLS above from the submandibular space below, and it lies superior to the anterior belly of the digastric muscle (Figure 1 and Figure 2d). This is a paired sling-like structure, originating from the mylohyoid ridge at the medial aspect of the body of the mandible and forming the muscular floor of the oral cavity. The bulk of the muscle fibres (anterior and middle) attach to the midline raphe, which extends from the mandibular symphysis to the hyoid bone and the posterior one-fourth of the fibres attach to the anterior surface of the hyoid bone.^1,6,7^

**FIGURE 3:**
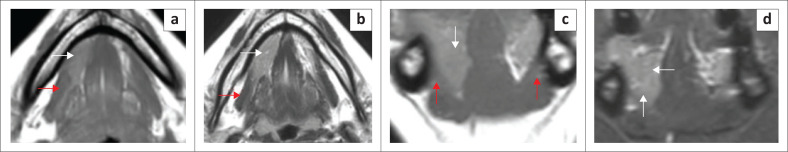
Mylohyoid Boutonniere in a 60-year-old patient who presented with intermittent swelling of the right submandibular region. T1 axial (a), T2 axial (b), T1 coronal (c) and T2-short tau inversion recovery (STIR) coronal (d) demonstrate a deficient right mylohyoid muscle (red arrow) anteriorly and an enlarged right sublingual gland herniating inferiorly through the mylohyoid muscle defect (white arrow).

**FIGURE 4:**
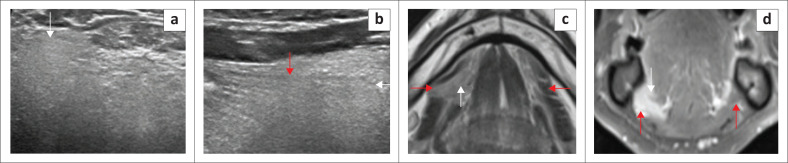
Mylohyoid Boutonniere in an adult patient who presented with a 2-year history of a fluctuating right submandibular neck lump. Ultrasound (a, b) demonstrate the enlarged inferiorly herniated right sublingual gland (white arrow) through the mylohyoid defect (red arrow). Subsequent MRI for ongoing concerns confirms the findings with a mylohyoid defect (red arrow) and herniated sublingual gland (white arrow) on the T2W axial image (c) and T1 contrast-enhanced fat-suppressed coronal image (d).

Defects of the mylohyoid muscle are a common, well-recognised entity, referred to as mylohyoid boutonnieres, with a reported presence of 20% – 77% in the literature.^6,7^ White et al.^7^ confirmed mylohyoid defects in 77% of their cases; bilateral defects were observed in 67% and unilateral defects in 33%. The herniated salivary tissue is mostly from the sublingual glands and less likely from the submandibular gland, and can present as a mass in 40% of cases, with possible recurrent inflammatory changes^6,7,9^ (Figure 3 and Figure 4). Other lesions that can potentially dissect through the mylohyoid defect into the submandibular space include plunging ranulas, abscesses and malignancies.^7,9^ When evaluating the FOM on imaging, it is vital to carefully assess the integrity of the mylohyoid muscles bilaterally, which is clearly visible on the axial and coronal images.

## Imaging

**FIGURE 5:**
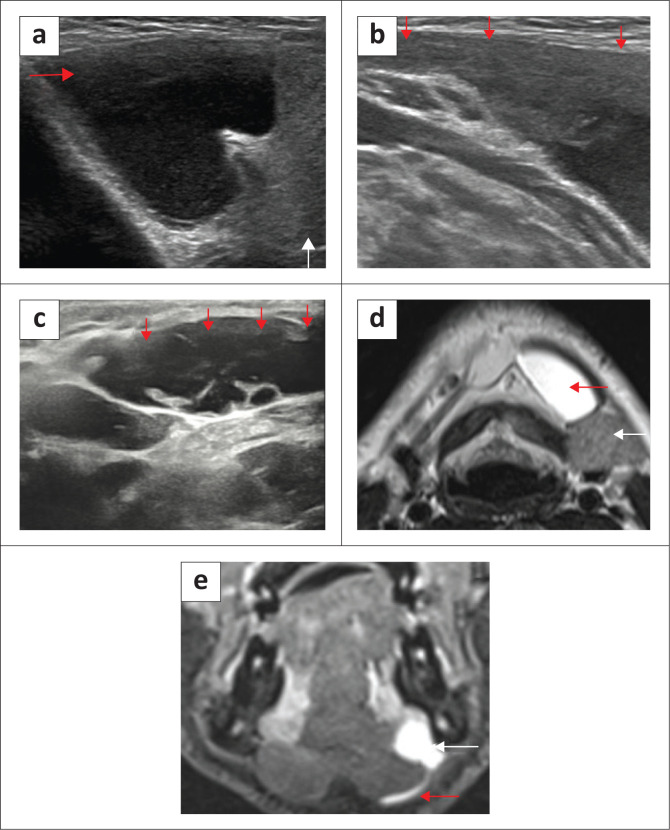
A case of a ranula in a 9-year-old child with a painless submandibular swelling. Ultrasound (a, b) showed a sonolucent cystic mass (4 cm × 2 cm; red arrows) with homogeneous low-level internal echoes, located medial to the submandibular gland (white arrow) and dissecting into the floor of the mouth. A different case of a ranula in an adult patient. Ultrasound (c) demonstrated a cystic mass, with internal debris and septations, in the left anterior submandibular region. MRI indicated the ranula (d, red arrow) on the T2W axial (d) and confirms extension to the sublingual space (e, white arrow) and communicating channel (e, red arrow).

Imaging is absolutely essential for the characterisation of various benign and malignant lesions.^1,2,3,4^ Ultrasound is a common first-line imaging modality to investigate neck abnormalities, with the advantages of easy availability, non-invasiveness, high-resolution imaging and lack of radiation. Ultrasound is the first modality of choice for the assessment of the neck lumps in the paediatric population. The major drawbacks of ultrasound are that the technique is operator-dependent, and it is limited in accurately assessing the extent of deep disease. Literature reviews highlight the role of ultrasound in evaluating the FOM and oral cavity, including the depth of invasion of tongue malignancies, beyond the standard practise of assessing the neck for the thyroid gland, salivary glands, superficial lumps and ultrasound-guided procedures.^1,10^ Figure 4 and Figure 5 highlight the role of ultrasound in demonstrating a myelohoid boutonniere and two cases of ranula.

CT and MRI are crucial complementary imaging modalities. MRI is an excellent technique for soft tissue characterisation, disease localisation, establishing early bone marrow infiltration and perineural spread of disease. CT is distinctly superior for investigating acute infections and abscesses, demonstrating benign and malignant cortical bony changes, confirming calcification and chondroid tumour matrix, and establishing the presence of ductal calculi.^3,11^ However, images of the oral cavity and FOM are prone to degradation by artefacts from dental amalgam and other post-surgical metal implants, especially with CT imaging.

^18^F-fluorodeoxyglucose positron emission tomography/computed tomography is an immensely important problem-solving complementary imaging technique to establish primary neck disease, detect nodal disease, distant metastasis, synchronous primary tumours and recurrent or residual disease. In the presence of post-treatment changes, diagnosing recurrent or residual disease is often a diagnostic challenge.

Other helpful imaging tools are conventional sialography for salivary ductal assessment and angiographic studies for specific arterial or arterio-venous vascular abnormalities.

## Floor of the mouth lesions

Most pathological abnormalities of the FOM are benign in nature, including developmental conditions (dermoid, epidermoid, thyroglossal cyst), ranula, lipoma, post-infective or inflammatory conditions caused by obstructive calculus disease of the submandibular duct with abscesses, sublingual adenitis and Ludwig Angina.^1,3,8,11^ The remaining infrequent benign lesions of the FOM are lymphatic and vascular malformations and rarely pleomorphic adenomas and benign neurogenic tumours. The mylohyoid Boutonniere lesions, seen as mylohyoid muscle defects with ectopic salivary gland tissue, are referred to as pseudolesions.^6,7^

The most common malignancies are squamous cell carcinoma (SCC), tumours of the salivary glands (mucoepidermoid carcinoma, adenoid cystic carcinoma) and lymphoma, which is the third most common malignant tumour.^1,3,4^

### Cystic lesions: Ranulas

Ranulas are benign mucus retention cysts of the FOM and submandibular regions that often develop in young people but can also be seen later in life. Because of the absence of true epithelial linings, they are not true cysts and develop as a result of blockage of the sublingual gland ducts (Rivinus duct). The underlying aetiology may be idiopathic or related to previous trauma or inflammation.^6,10^

**FIGURE 6:**
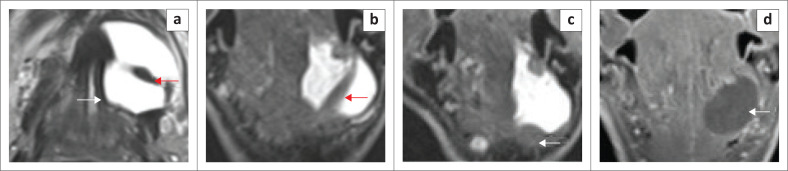
A ranula in an 85-year-old patient who presented with a painless swelling in the left floor of the mouth. MRI neck T2-weighted (T2W) axial (a) T2-weighted short-tau inversion recovery (T2W-STIR) posterior coronal image (b) reveal the left floor of the mouth simple cystic mass dissecting through the left mylohyoid muscle (red arrow) and indenting the left geniohyoid muscle (a, white arrow). The anterior coronal T2W short-tau inversion recovery (STIR) image (c) shows the cyst limited by the anterior belly of digastric muscle inferiorly (white arrow). No enhancement of the cyst wall or intracystic enhancing component on the contrast-enhanced coronal T1 fat-suppressed image (d).

**FIGURE 7:**
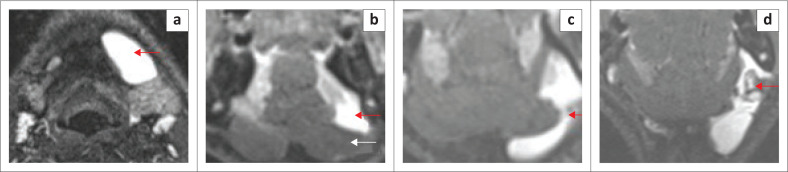
A plunging (diving) ranula in an adult patient with a history of a fluctuant soft swelling in the left submandibular region. Initial ultrasound (not included) revealed a partly imaged cystic lesion. T2 axial MRI neck (a) indicates a cystic lesion in the left submandibular region, anterior to the left submandibular gland, extending from anterior floor of the mouth. The T2-weighted short tau inversion recovery (T2W-STIR) coronal anterior image (b) shows the anterior extent of the cyst in the left sublingual space (red arrow) above the anterior belly of digastric muscle (b, white arrow). The mid part of lesion (c) shows the cyst is dissecting inferiorly, deep to mylohyoid muscle (red arrow), suggesting a tail-sign. The T2-weighted short-tau inversion recovery (T2W-STIR) coronal image (d) shows small non-cystic debris (red arrow); there was no evidence of a vascular malformation.

Ranulas are broadly classified into two categories: simple ranulas are confined to the SLS, bordered inferiorly by the mylohyoid muscles (Figure 6); while the plunging (diving) variety of ranula dissects into the inferior submandibular space through the communication posterior to the mylohyoid muscle or dissects through the mylohyoid muscles^9,10^ (Figure 7).

Ranulas can present as an intraoral mass or as a cystic, fluctuant, submandibular mass of varying size. The role of imaging is to establish the anatomical location (sublingual or plunging variety), to demonstrate the benign cystic nature of the lesion, to differentiate it from other cystic lesions of the FOM (lymphangioma, epidermoid, dermoid cyst and branchial cleft cysts), to look for integrity of the mylohyoid muscles and above all, for pre-surgical assessment with the aim of complete excision of the sublingual gland.^7,10^

Ultrasound is the initial imaging of choice, particularly in the paediatric age group. An uncomplicated ranula appears as a unilocular, anechoic cystic lesion, while complicated or chronic haemorrhagic ranulas demostrate varying degrees of debris and wall-thickening^9,12^ (Figure 5). Ultrasound may have a limited role in large plunging ranulas or suspected ranulas with indeterminate features. In evaluating plunging ranulas, CT and MRI can better demonstrate the site of communication between the sublingual and submandibular spaces posterior to the mylohyoid, where the communicating narrowed cystic tract is seen directed anteriorly. This appearance is referred to as the ‘tail sign’, which is diagnostic of plunging ranula^10,13^ (Figure 5e and Figure 7c).

### Cystic lesions: Developmental

The squamous epithelium lined epidermoid and dermoid cysts and cystic teratomas of the neck are rare developmental entities, mostly seen at the FOM as slow-growing midline cysts and constitute only 1.6% to 6.9% of head and neck cystic masses.^14,15,16^ Ultrasound, CT and MRI can confirm the cystic nature of the lesion; however, MRI is the most sensitive modality for further characterisation and aids in differentiation from other cystic sublingual, submandibular and submental masses like ranula, cystic hygroma, lymphangioma and also, rarely, abscesses in cases of infected cysts.^15^

The ectoderm-lined dermoid cyst demonstrates the diagnostic features of an intracystic cluster of floating fatty corpuscles, known as the ‘sack of marbles’ sign, which on CT appears as a cluster of low attenuating densities and on MRI, demonstrates typical features of fat content.^16^

**FIGURE 8:**
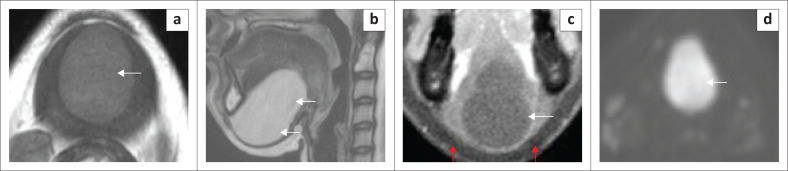
Epidermoid cyst in an adult female patient. T1 axial MRI neck with contrast (a) shows a large well defined, 5 cm, simple, non-enhancing cystic mass at the floor of mouth (a, white arrow) with intermediate homogeneous signal, splaying the anterior belly of the digastric muscles. T2 sagittal (b) shows homogeneous hyperintensity (white arrows). The contrast-enhanced T1 fat-saturated coronal image (c) shows a non-enhancing cyst (white arrow), limited inferiorly by an intact mylohyoid sling (red arrows). There is a marked restriction of diffusion on the high b-value diffusion-weighted image (d).

The simple, squamous epithelium lined epidermoid cyst typically appears as a midline FOM, simple, fluid-containing cyst on imaging and can attain a very large size because of slow growth^14,17^ (Figure 8). Restriction of diffusion is the most characteristic feature of epidermoids on MRI (Figure 8d), differentiating it from the dermoid cyst.

The most common congenital lesions of the neck are thyroglossal duct cysts, which are rarely located in the FOM.^18^ The majority of these cysts are infrahyoid in location, with the suprahyoid site being less common, seen in only 20% of cases. A characteristic thyroglossal cyst appears as a painless, midline cystic mass in the paediatric population, the paramedian location is more common with the infrahyoid cysts. Ultrasound is the initial imaging of choice, which will show a simple, uncomplicated cyst as an anechoic, thin-walled cyst without internal solid components or vascularity. However, a more complex appearance on ultrasound would be seen in the presence of infection, haemorrhagic contents, or the rare coexistence of thyroid carcinoma within the cyst. Further imaging with CT or MR imaging would be essential to characterise more complex lesions, to demonstrate the extent of the deeper, larger lesions and for pre-surgical planning.

### Floor of the mouth infection/inflammation

**FIGURE 9:**
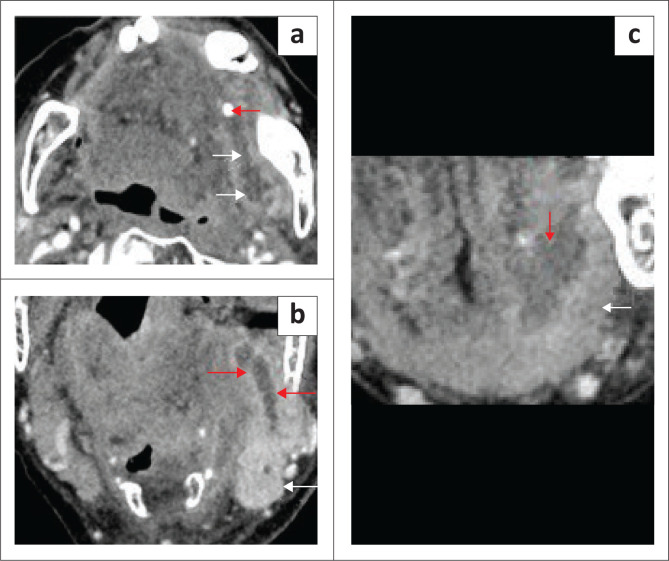
(a, c) Obstructing submandibular duct calculus with adenitis and a floor of the mouth (FOM) abscess. A 98-year-old female patient presented with a large, tense swelling in the left FOM and submandibular region, a swollen tongue and difficult breathing. Contrast-enhanced axial CT neck (a) demonstrated a calcified obstructing calculus at the distal end of the left submandibular duct (red arrow) and a markedly dilated duct (white arrows). The left submandibular gland was enlarged and markedly enhancing because of acute adenitis (b, white arrow and dilated duct, red arrows). There is a left anterior FOM collection (c, red arrow), deep to the left mylohyoid muscle (c, white arrow).

Acute infection of various neck spaces, including the FOM, is often associated with significant morbidity and mortality, particularly in elderly, diabetic and immunocompromised patients. The major underlying factors for acute FOM infections (cellulitis and abscesses) are related to salivary ductal calculus disease or odontogenic causes.^8^ The submandibular (Wharton) duct is a common site for intraductal calculi with the potential of causing post-obstructive suppurative adenitis and abscesses (Figure 9). Through the free anatomical communication posteriorly, cellulitis from an infected submandibular gland carries the risk of extension into the SLS and vice versa.^8,11^

**FIGURE 10:**
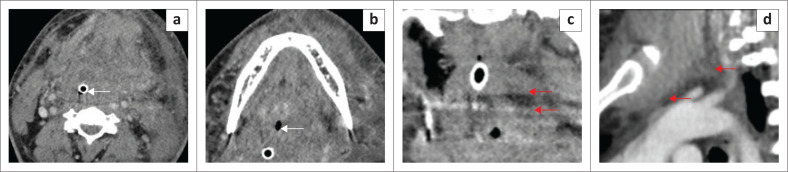
A case of Ludwig’s Angina in an adult patient who presented with acute stridor, difficulty swallowing and marked swelling in the left upper neck. The patient was intubated. CT neck with contrast was partly degraded by artefacts from dental amalgam. There was extensive soft tissue thickening of the floor of the mouth (FOM) (a, b) with the loss of the normal anatomical boundaries at the FOM and other neck spaces, marked narrowing of airspaces (b, white arrow) and evidence of intubation (a, white arrow). There was an irregular left parapharyngeal abscess (c, red arrows), which was drained. The sagittal image (d) shows the extension of cellulitis into the anterior mediastinum (red arrows).

The FOM is also involved in other acute infections of the neck spaces, like Ludwig’s angina, which is a serious progressive acute bacterial infection, originating commonly from mandibular molar tooth infection. This disease entity is more common in diabetics and immunocompromised patients, and the infection can progress rapidly, causing airway compromise. Imaging demonstrates extensive cellulitis, predominantly involving the submandibular, submental and SLSs with a lesser likelihood of underlying abscess formation,^11,19^ as demonstrated in Figure 10.

**FIGURE 11:**
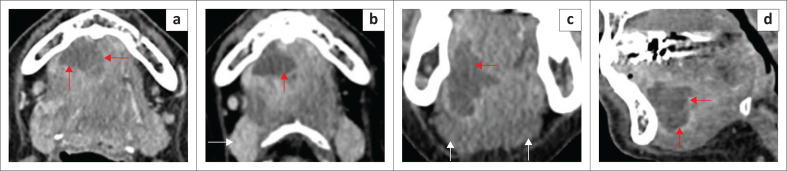
A 69-year-old male patient presented with a painful swelling in the right sublingual space, difficulty in swallowing and pain in the submandibular region. Contrast-enhanced CT (a to d) demonstrates an irregular rim-enhancing abscess (red arrows). The axial image (b) shows mild borderline enlargement with increased enhancement of the right submandibular gland (white arrow). The abscess is located above the mylohyoid muscle (c, white arrows).

Ultrasound is helpful for the assessment of superficial abscesses in the submandibular region, nodal disease and duct calculi. In acute settings, contrast-enhanced CT neck (from the base of the skull to the superior mediastinum) is the imaging modality of choice for confirmation and localisation of the abscesses, which appear as rim-enhancing fluid collections (Figure 11), pre-surgical planning, assessment of the patency of the major neck vessels and evaluation of the bony mandible for osteomyelitis.^11,19,20^ Recent studies have, however, also established the role of MRI in deep neck infection as a more accurate imaging modality because of its high soft tissue resolution.^20^ The major limitation is the availability of MR imaging in emergency settings.

### Floor of the mouth malignancy

Squamous cell carcinoma is the most common malignancy of the oral cavity (more than 90%), most frequently involving the oral tongue and mucosal lip, followed by the floor of mouth (17% of oral cavity SCC). Other malignant conditions affecting the FOM are the minor salivary gland tumours (mucoepidermoid carcinoma, low-grade adenocarcinoma and adenoid cystic carcinoma), lymphoma and rarely, sarcomas.^1,2,21,22^

**FIGURE 12:**
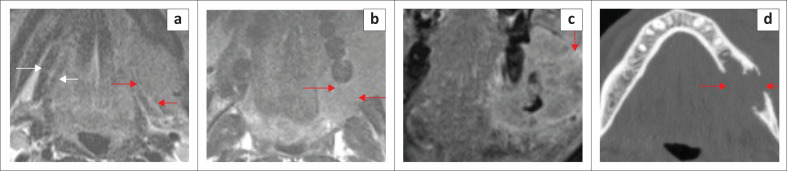
Stage T4 squamous cell carcinoma of the mandible in an adult patient. There is a large bulky mass lesion involving the left mandibular body, with full-thickness osseous destruction, and the lateral floor of the mouth, with invasion of the left mylohyoid and hyoglossus muscles (red arrows on the left and normal muscles on the right (white arrows) in (a, b; T2-axial views) and on the coronal T1 contrast-enhanced fat-suppressed image (c). There is extensive infiltration of the left masseter muscle (c) and retromolar trigone (b, red arrows). CT mandible (d) shows full-thickness cortical osseous destruction on the left.

**FIGURE 13:**
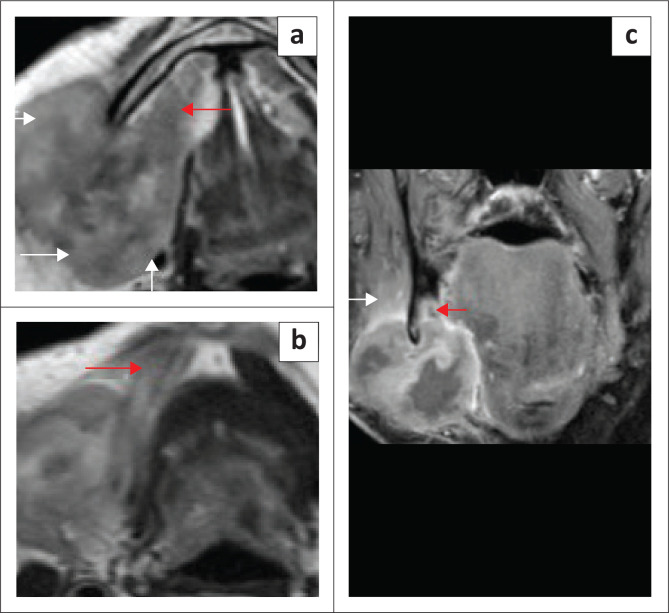
(a, b) Locally advanced squamous cell carcinoma, seen as a right submandibular gland-based aggressive mass, invading the floor of the mouth. Axial T2 (a) shows the large tumour (white arrows) and invasion of right sublingual gland (red arrow). Axial T2 (b) demonstrates invasion of the entire anterior belly of right digastric muscle (red arrow). The T1 contrast-enhanced fat-saturated coronal image (c) shows the heterogeneously enhancing mass with necrotic areas. There is focal invasion of bony mandible (red arrow) and inferior attachment of right masseter muscle (white arrow).

Malignancies of the mandible and submandibular gland can also invade the FOM,^1,21^ as demonstrated in Figure 12 (T4 SCC mandible invading the FOM) and Figure 13 (SCC of submandibular gland infiltrating the FOM). Malignancies of the FOM spread further by invading the muscles of the FOM, the mandible, the musculature of tongue, the submucosal spaces and extending along the neurovascular structures in the SLS.

CT and MRI are complementary imaging techniques to characterise and evaluate the extent of disease. MRI is an excellent technique and imaging of choice, with particular reference to the assessment of the depth of tumour invasion, which is a crucial prognostic marker for T-staging of oral cavity cancers.^23,24^ MRI is also very sensitive for detecting early bone marrow infiltration and establishing perineural spread of disease, which is particularly a concern for adenoid cystic cancers.^22,23,25^

According to the revised T-staging guidelines of the American Joint Committee on Cancer (AJCC) 2018,^26^ depth of invasion and tumour size are the criteria for T1 to T3 staging, full-thickness osseous destruction of the mandible and maxilla for T4a staging and invasion of the masticator spaces and skull base, and encasement of the internal carotid arteries for T4b staging (Box 1).^26^ Imaging of the superficial, smaller, mucosal T1 tumours poses diagnostic challenges where clinical inspection and biopsy are superior in assessment. However, MRI remains excellent in establishing spread to the tongue and FOM musculature, ductal obstruction and bone marrow invasion.

BOX 1 T-staging of the oral cavity cancers.

**Table:** 

TX	Primary tumour can not be assessed.
Tis	Carcinoma in-situ.
T1	Tumour ≤ 2 cm and DOI ≤ 5 mm.
T2	Tumour ≤ 2 cm and DOI > 5 mm and ≤ 10 mm, or Tumour > 2 cm and ≤ 4 cm and DOI ≤ 10 mm.
T3	Any tumour with DOI > 10 mm, or Tumour > 4 cm with DOI ≤ 10 mm.
T4a	Tumour > 4 cm with DOI > 10 mm, or Tumour invades adjacent structures only (e.g. through cortical bone of mandible or maxilla), involves the maxillary sinus or skin of the face. FOM & Extrinsic muscles of tongue.
T4b	Tumour invades masticator space, pterygoid plates, or skull base and/or encases the internal carotid artery.

*Source:* Zanoni DK, Patel SG, Shah JP. Changes in the 8th edition of the American Joint Committee on Cancer (AJCC) staging of head and neck cancer: Rationale and implications. Curr Oncol Rep. 2019;21(6):52. https://doi.org/10.1007%2Fs11912-019-0799-x

DOI, depth of invasion; FOM, floor of the mouth; T, T-staging.

**FIGURE 14:**
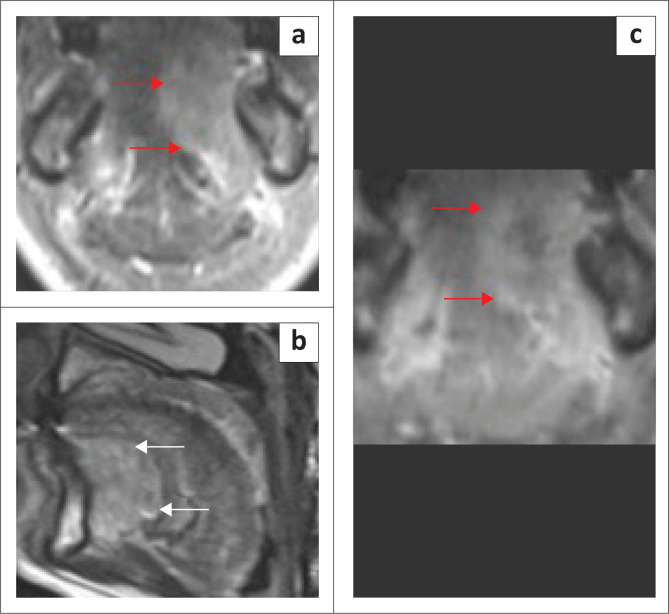
Left anterior floor of the mouth bulky small cell carcinoma (stage T4) in an elderly patient, invading the extrinsic tongue muscles. T2-weighted short-tau inversion recovery (T2W-STIR) coronal (a) and sagittal T2 (b) demonstrate the mildly hyperintense, bulky tumour. The T1 contrast-enhanced fat-suppressed image (c) reveals an ill-defined, inhomogeneously enhancing tumour.

**FIGURE 15:**
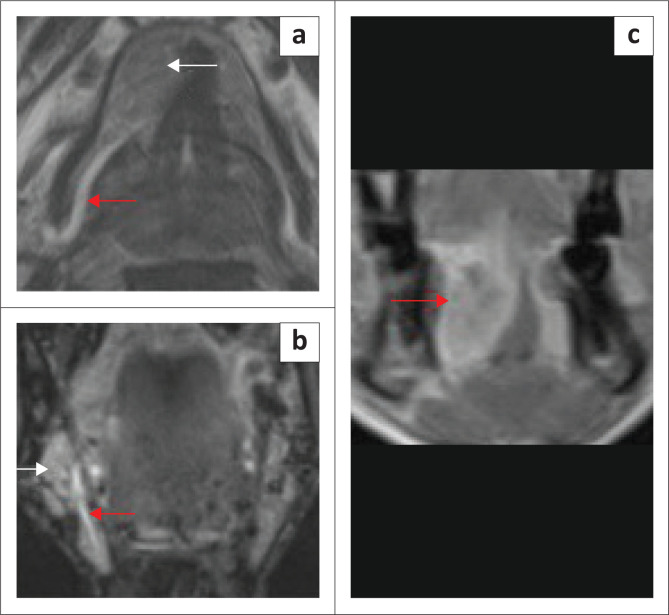
Stage T4 anterior floor of the mouth (FOM) locally advanced squamous cell carcinoma in a 58-year-old male patient who presented with recurrent swelling in the right submandibular region and fullness in the FOM. Axial T2 (a) shows the dilated right submandibular duct (red arrow) and the right FOM tumour obstructing the duct (white arrow). The coronal short tau inversion recovery (STIR) (b) shows the oedematous right submandibular gland (white arrow) and dilated duct (red arrow). The coronal contrast-enhanced fat-suppressed image (c) demonstrates the inhomogeneously enhancing tumour invading the extrinsic tongue muscles and right sublingual gland (red arrow).

**FIGURE 16:**
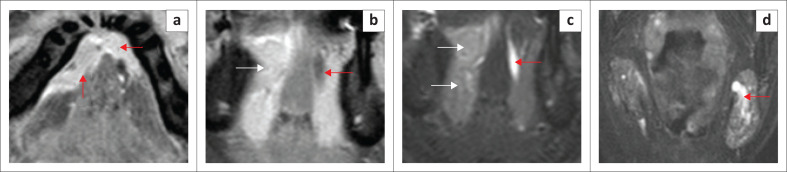
Stage T2 squamous cell carcinoma of the right floor of the mouth with enlargement of the right sublingual gland, crossing the midline and obstructing the left submandibular duct in a 68-year-old male. The T1 post-contrast fat-suppressed axial image (a) shows the tumour crossing the midline (red arrows). The T1 post-contrast fat-suppressed coronal image (b) demonstrates the less enhancing tumour component (white arrow) in contrast to the normal sublingual gland and the dilated left submandibular gland duct (red arrow). The T2-weighted short tau inversion recovery (T2W-STIR) coronal image (c) shows the tumour involving the right sublingual gland (white arrows) and a dilated distal left submandibular gland duct (red arrow). The T2-STIR coronal image (d) shows the enlarged left submandibular gland with a dilated ductal system (red arrow).

**FIGURE 17:**
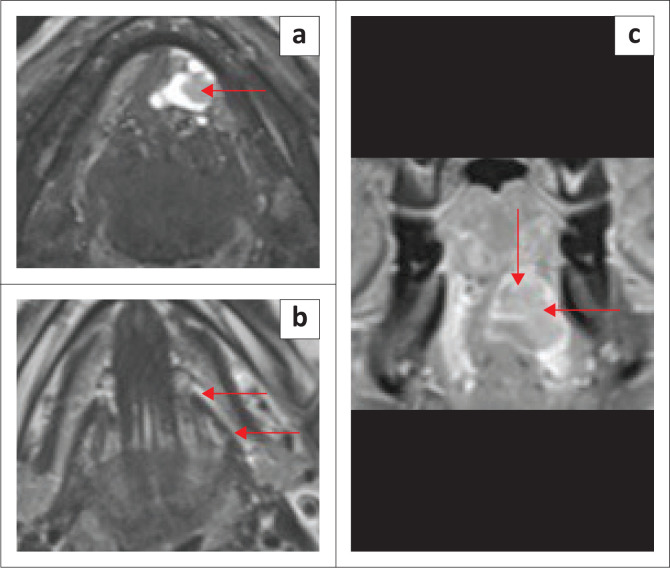
Mucoepidermoid carcinoma of the salivary gland in the left anterior floor of mouth/sublingual space, in an adult patient. A tumour is obstructing the opening of left submandibular duct. T2-weighted short tau inversion recovery (T2W-STIR) axial image (a) shows the slightly hyperintense tumour projecting into left submandibular gland duct opening (red arrow). The T2 axial (b, red arrows) shows the dilated left submandibular gland duct. The T1 contrast-enhanced fat-suppressed coronal image (c) demonstrates the enhancing tumour (red arrows).

On imaging, SCC tumours typically appear as poorly defined, heterogeneously enhancing masses, most likely with cystic and necrotic components (Figure 14, Figure 15 and Figure 16). Figure 13 demonstrates a rare case of primary SCC of the submandibular gland with invasion of the muscles of the FOM and bony invasion of the mandible. Patients can present with features of recurrent sialadenitis secondary to submandibular duct obstruction at the FOM (Figure 16 and Figure 17). The most common condition mimicking malignancies of the FOM is infection of the oral cavity with associated cellulitis or abscess.^21,22,25^

Lymphomas contribute 14% of head and neck malignancies and the vast majority are non-Hodgkin lymphomas (96%). Lymphoma of the oral cavity is generally uncommon and constitutes only 5% of oral malignancies. Primary non-Hodgkin lymphoma of the mandible is extremely rare, accounting for only 0.6% of extranodal lymphomas and 8% of mandibular malignancies.^27,28^

**FIGURE 18:**
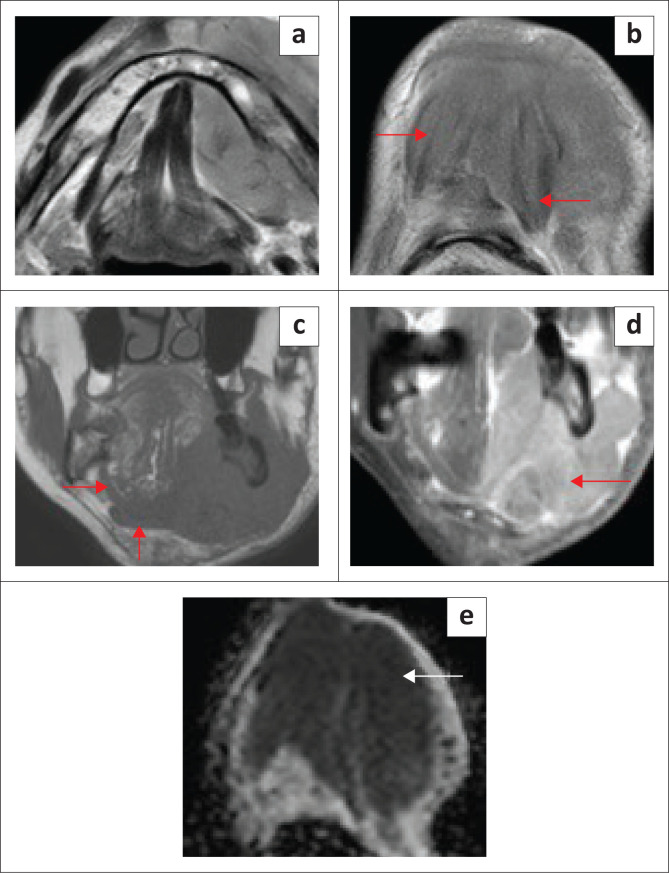
An 89-year-old male patient with aggressive diffuse large B-cell lymphoma. MRI neck; T2 axial (a, b), unenhanced T1 coronal (c) and post-contrast T1 fat sat coronal (d) demonstrate a large bulky soft tissue mass with full-thickness marrow infiltration and cortical destruction of the left mandibular body. The mass is involving the left floor of the mouth (FOM) and sublingual space with infiltration of the left muscles of the FOM, submental region and left submandibular gland. There is a marked restriction of diffusion on the apparent diffusion coefficient (ADC) map (e). T1 coronal (c) shows normal right mylohyoid and anterior belly of digastric muscles (red arrows).

At imaging, these rare FOM lymphomas present as characteristically bulky, homogeneous soft tissue masses with a varying degree of significant contrast enhancement and pronounced restriction of diffusion. The osseous abnormalities can appear as ill-defined lytic destruction or an infiltrative pattern with invasion and widening of the inferior alveolar nerve canal. Typical radiological findings are presented in Figure 18.

## Post-treatment imaging

Interpretation of the imaging findings in the presence of post-treatment changes is often a diagnostic challenge. It is crucial for the radiologist to be aware of the time gap between the completion of treatment and imaging, as follow-up imaging is performed at least 3 months after the completion of the treatment and significant imaging variations may be present because of radiotherapy-induced oedematous changes. Furthermore, it is crucial to understand the various types of surgical procedures (type of graft repair, implants and neck dissection) that impact the imaging findings.^29^

**FIGURE 19:**
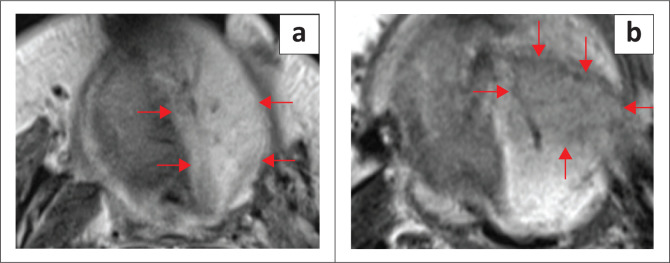
An elderly patient with tumour recurrence within the graft in the left floor of the mouth (FOM). Axial T2 images (a, b) show the homogeneous signal of the graft (a, red arrows) and recurrence within the graft with a loss of the bright signal (b, red arrows).

**FIGURE 20:**
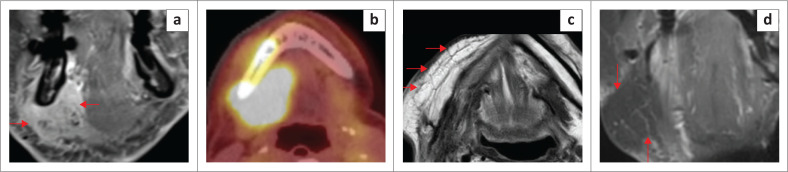
An elderly patient with a malignant tumour in the right FOM and submandibular region as seen on the T1 coronal post contrast (a). The lesion is markedly avid on ^18^F-fluorodeoxyglucose positron emission tomography/computed tomography (^18^F-FDG PET/CT) (b). The T2 axial (c) shows the post-operative appearance after right mandibular resection and graft repair (bright signal of graft fat; red arrows). The contrast-enhanced fat-suppressed T1 weighted (T1W) coronal image (d) shows complete suppression of fat signal (red arrows) and mild oedema at the surgical site.

**FIGURE 21:**
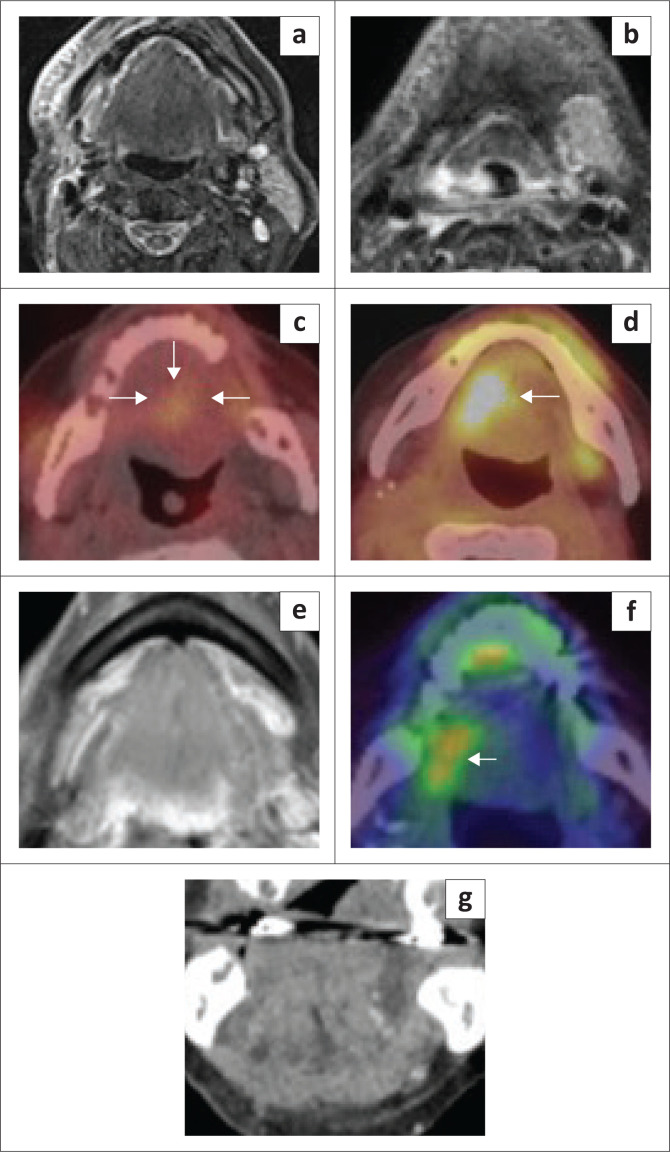
False-positive uptake on positron emission tomography (PET) imaging. An elderly female patient who underwent resection of an oral tongue squamous cell carcinoma and neck dissection, followed by radiotherapy. The baseline post-treatment T2 axial images (a, b) show marked oedema in the right upper neck and floor of the mouth (FOM) region with an absent right submandibular gland and oedematous left submandibular gland. A ^18^F-fluorodeoxyglucose positron emission tomography/computed tomography (^18^F-FDG PET/CT) (c) performed after 6 months for a synchronous sinister lung lesion showed non-significant uptake at the FOM. A follow-up ^18^F-FDG PET/CT (d) performed for a malignant lung lesion after 1 year showed significant uptake at the right FOM; however, the MRI (e, post-contrast fat-suppressed axial) and clinical examination did not reveal any mass lesion at the FOM. Persistent PET uptake after another year (f) and normal FOM on coronal contrast-enhanced CT neck (g).

MR imaging can pose a significant challenge in the differentiation of recurrent or residual disease from post-treatment changes. Although ^18^F-FDG PET/CT has a very high negative predictive value for excluding recurrent disease in comparison to CT and MRI (Figure 19 and Figure 20), PET imaging also has its own limitations, including the possibility of false-positive results from post-treatment changes and physiological causes,^5,29^ as illustrated in Figure 21.

## Conclusion

Precise understanding of the anatomical landmarks of the FOM and imaging findings of various benign and malignant conditions is crucial for diagnosis and assessment of the pathways of disease spread, T-staging of cancers, treatment planning and disease surveillance.
